# Including patients in core outcome set development: issues to consider based on three workshops with around 100 international delegates

**DOI:** 10.1186/s40900-016-0039-6

**Published:** 2016-07-08

**Authors:** Bridget Young, Heather Bagley

**Affiliations:** 1 Institute of Psychology Health and Society / North West Hub for Trials Methodology Research, University of Liverpool, Brownlow Hill, Liverpool, L69 3GB United Kingdom; 2 Clinical Trials Research Centre / The COMET Initiative, Alder Hey Children’s NHS Foundation Trust, Institute of Child Health, University of Liverpool, Eaton Road, Liverpool, L12 2AP United Kingdom

**Keywords:** Core outcome sets, Patient and public involvement, Consensus, Patient engagement, Public engagement

## Abstract

**Plain English summary:**

This commentary article describes three interactive workshops that explored how patients can contribute to decisions about what outcomes are measured in clinical trials across the world. Outcomes like quality of life, side-effects and pain are used in trials to measure whether a treatment is effective. Here, we outline how research groups are increasingly coming together to develop ‘core outcomes sets’ for particular conditions. Core outcome sets are lists of agreed outcomes. Their use will help in identifying which treatments are effective by enabling people to compare the findings of different clinical trials in the same condition. Currently, it is often very difficult to make these comparisons because different studies often measure different outcomes. Delegates attending the workshops included patients, clinicians and researchers. They discussed ways of making core outcome set development more meaningful and accessible for patients, and ensuring that they have a genuine say in the development process. This article summarises these discussions and concludes by identifying three distinctive challenges in securing patient input to core outcome set development: the process and objectives can seem far removed from the immediate concerns of patients, difficulties can arise in securing patient input on an international scale, and difficulties can also arise in bringing multiple stakeholder groups together to achieve consensus. While patient participation, involvement and engagement in core outcome set development can draw on lessons from other research areas, these distinctive challenges point to the need for distinctive solutions to enable meaningful patient input to core outcome set development.

**Abstract:**

**Background** This article describes three workshops that explored how patients can contribute to decisions about what outcomes are measured in clinical trials. People need evidence about what treatments are best for particular health conditions. The strongest evidence comes from systematic reviews comparing outcomes across different studies of treatments for a particular condition. However, it is often difficult to do these comparisons because the different studies—even though they have all investigated the same condition—often measure different outcomes. To tackle this problem, research teams are increasingly coming together to develop core outcome sets (COS) for particular conditions or treatments. The goal is that across the world, all the research teams working on the same condition or treatment will then use the COS in their research.

**Main body** We report on three interactive workshops that explored how patients and the public can contribute to decision making about what outcomes should be included in a COS. About 100 international delegates, including researchers, clinicians and patients, attended the workshops. The workshops were held in the United Kingdom, Italy and Canada as part of the COMET (Core Outcome Measures in Effectiveness Trials) Initiative annual meetings. Patients who had some experience as research advisors, collaborators, partners or co-ordinators facilitated the workshops together with a researcher. Notes made during each workshop informed the preparation of this article. Workshop discussion focussed on ways of making core outcome set development more meaningful and accessible for patients. Delegates wanted patients to have a genuine say, alongside other stakeholders, in what outcomes are included in COS. Delegates felt that key to ensuring this is recognising that patient participation in COS development alone is not enough, and that patients will also need to be involved in the design of COS development studies.

**Conclusion** We conclude by pointing to some distinctive challenges in including patients in COS development. While the COS development community can draw on the lessons learnt from other research areas about patient participation, involvement and engagement, the distinctive challenges that arise in COS development point to the need for some distinctive solutions too.

## Background

### What are core outcome sets and why are these needed?

In choosing between different treatments, patients, the public, health professionals and other stakeholders need evidence about what works. Researchers and clinicians get this evidence by carrying out studies to measure the effects that different illnesses, conditions and treatments have on patients. In research contexts these effects are often called ‘outcomes’. Outcomes can include a wide range of effects like, quality of life, side-effects, days off work, fatigue, pain etc. The specific outcomes that researchers measure will depend on the patient group, illness and treatment being studied. Imagine if you wanted to know what works best for treating back pain. The strongest evidence would come from comparing outcomes from all high quality studies of back pain treatments. But the treatments can only be compared if the research teams have measured at least some outcomes ‘in common’ across the different studies. Instead, what currently tends to happen is that there may be dozens of studies looking at a particular condition, but only a few will have measured more than a handful of outcomes in common. In some cases there may be only one or two consistent outcomes across the studies [1]. A recent example is a systematic review (see glossary of terms in Table 1) of exercise for patients with depression [2]. The review found that 37 clinical trials had measured depression, but only six of the trials had measured adverse effects (e.g. pain, fatigue), 3 had measured quality of life and none of the trials had measured the costs involved. This is wasteful [3, 4] and makes it difficult or impossible to know what treatments are best, as the outcomes cannot be compared across different studies or in systematic reviews.

**Table 1:** Glossary of terms

Systematic reviews – “A systematic review summarises the results of available carefully designed healthcare studies (controlled trials) and provides a high level of evidence on the effectiveness of healthcare interventions. Judgments may be made about the evidence and inform recommendations for healthcare” [22]. Qualitative studies – “Qualitative research is used to explore and understand people’s beliefs, experiences or behaviours. It asks questions about how and why. Researchers use methods like focus groups and interviews” [23]. Consensus processes – Consensus processes are surveys, meetings and discussions where the opinions of relevant experts are drawn together to try to reach agreement, or “consensus” about a particular topic. Experts can include patients and carers with experience of a condition. The process of reaching consensus can involve methods like surveys and consensus meetings. Delphi surveys – A method to gather the opinions of a group of experts on a subject and try to reach agreement, or “consensus” amongst this group about a particular topic. Experts can include patients or carers with experience of a condition. Delphi surveys are usually questionnaires and can involve the experts voting to rate their opinion on a topic in two or more rounds of the survey. Plain language summaries about Delphis [24] and core outcome set studies [6] are available from the COMET website. PoPPIE Working Group – PoPPIE stands for People and Public Participation, Involvement and Engagement. PoPPIE’s international membership will be leading COMET’s public participation, involvement and engagement activities. The COMET Initiative – COMET stands for the ‘Core Outcome Measures in Effectiveness Trials’ Initiative. It involves people from around the world and in many different areas of health and social care and was set up to help in two main ways: • to provide the COMET database as a central point where researchers, clinicians and patients can find core outcome sets that have already been developed or are still under development. • Provide materials to support the teams who are working on core outcome sets	

In response to this problem, research teams have started to develop core outcome sets (COS) [5]. In essence, COS are just lists of outcomes-albeit very important lists. The key idea is that when a COS is developed, all the research teams working on the same illness, condition or treatment will then use that COS in their research. There are several other special things about COS:

- COS should only include outcomes that are fundamental (i.e. core) to a certain condition. This leaves research teams free to measure additional outcomes in their trials if they wish.
- Different stakeholders should reach a consensus about what outcomes go in the COS. Developing a COS is not as easy as it might sound. There may be dozens of important outcomes, but relatively few should be included in one COS. Having lots of outcomes in a COS would be a burden for patients in future studies, make the research expensive and deter researchers from using COS.
- The process of agreeing what outcomes should be in (or out) of a COS needs to be fair, transparent and inclusive of people from diverse backgrounds.
- COS need to be relevant across different countries. COS will be of little help if researchers in only one or two countries use them, but if used widely, COS may profoundly improve the power of research to benefit patients.
- While we focus in this article on the issue of ‘what’ outcomes should go in a COS, a related issue is ‘how’ to measure those outcomes (i.e. which tools or questionnaires should be used to measure effects such as pain, fatigue etc., and at which time intervals should the measures be taken). Some COS developers tackle both issues in the same project. However, deciding what to measure tends to be the first step in the process [1] and so that was the focus of our workshops.

A plain language summary on COS can be found on the website of the Core Outcome Measures in Effectiveness Trials Initiative [6] (COMET-see glossary of terms in Table 1).

### Why include patients in COS development?

In deciding what outcomes should be included or be left out of a COS, people who know what it’s like to live with the effects of conditions and treatments should have a say. The people who usually know this best are patients and their families or carers (caregivers in North America). The views of the wider public on what outcomes should go in a COS are sometimes important too. For example, many interventions such as healthy lifestyle promotion or vaccines are implemented in community settings rather than in healthcare settings, or are intended for people for whom the term ‘patient’ is not appropriate (e.g. disabled people or users of mental health services). However, for brevity we refer mainly to ‘patients’ in this article.

If patients do not have a say in their development, there is a likelihood that COS will omit important outcomes, and ultimately, that the research will fail to give definitive information about whether treatments benefit patients or not. There are examples where patients have identified outcomes important to them that researchers and clinicians had previously overlooked [7] or saw as being of little importance [8]. COS developers have limited experience of working with patients to identify what outcomes matter most to them [5]. Developers cannot assume the methods that work for seeking the input of professionals will also be suitable for patients. Additionally, relatively little is known about the sort of input that patients have had in COS development so far and whether they really are having a say in what outcomes get included [9].

### A note on terminology

In the remainder of this article we discuss both patient *participation* in COS development studies and patient and public *involvement* in these studies. Patient participation is where patients and the public take part in a COS development study, for example alongside clinicians and other stakeholders in a consensus survey, and give data on their opinions regarding what outcomes are important. We refer to the people in this role as ‘patient participants’ or ‘participants’. Patients and the members of the public may also be involved in designing a COS development study, overseeing it once it is running and helping with its dissemination, a process referred to in the UK as patient and public involvement or PPI. We refer to the people who take these roles as ‘patient research partners’ or ‘partners’, although others may use terms such as patient ‘advocates’, ‘representatives’, ‘contributors’, ‘surrogates’ or ‘community stakeholders’ etc. Where it is necessary to refer to patients having a role in COS development generally, we refer to them having input or being included in COS development.

### What methods have been used to develop COS?

COS development tends to take place over several stages, involving methods such as systematic reviews, qualitative studies and consensus processes [5] (see glossary of terms in Table 1). As in any other research activity, patients can input to COS studies as participants or research partners during all of these stages. However, recent reviews [5, 9] indicate that few of the COS studies published to date have had any patient input, although there are indications of a marked increase in the number of *ongoing* COS studies that include patients. In published studies that have had some form of patient input, many do not clearly distinguish between patient participation and patient involvement in COS development. Additionally, from what we can discern, patient input in published studies has largely been limited to patient participation in the consensus stages of COS development. Nevertheless, there are some published studies that have had input from patients at an earlier stage in the COS development process, while ongoing studies may increasingly be doing likewise [10–14].

**Fig. 1:**
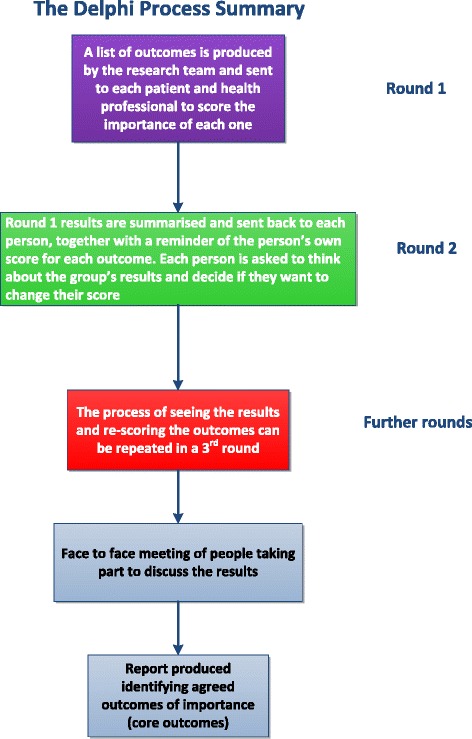
Flow chart summarising a Delphi survey or process

Systematic reviews are often carried out at an early stage in the COS development process [1]. These usually involve identifying what outcomes researchers and clinicians have previously thought important to measure based on their experience. COS developers will use the systematic review findings to develop an initial ‘long list’ of outcomes to go forward to the next stage of COS development. Occasionally, qualitative studies, comprising individual interviews or focus groups are used to complement the systematic review findings by identifying what outcomes patients and other stakeholders think are important [10, 15]. The consensus development stage may come next. At this stage COS developers draw together different stakeholders (ideally including patients) to collect, compare and discuss opinions, and then reach a final agreement on what outcomes should be in a COS. An increasingly common step in reaching a consensus is to carry out a Delphi survey [9] (see glossary of terms in Table 1 and Fig. 1). Delphi surveys can be carried out online, by post, or telephone and usually involve 2–3 rounds. The process is anonymous to make sure everyone has an equal say. In round one, participants rate the importance of each outcome from the long list. In the second round the participants receive a summary of the group’s ratings last time and a reminder of their own ratings. They will be asked to reflect on these and rate each outcome again. While participants do not have to change their ratings, the hope is that the ratings in the second or subsequent rounds will show more agreement regarding which outcomes are felt to be the most important. After the Delphi survey, COS developers often invite the Delphi participants and other stakeholders to meet face-to-face to review the Delphi findings, and discuss and vote on the outcomes to reach a final consensus. Some COS developers work towards consensus development without Delphis, relying on face-to-face meetings, structured discussions and voting to reach consensus.

## Main text

### Why were workshops on including patients in COS development needed?

As noted above, we are aware that patients are increasingly being included in ongoing COS development studies particularly as participants [9]. But less is known about the best methods for doing this. We therefore decided to run some workshops with delegates and patients at meetings of the COMET Initiative (see glossary of terms in Table 1). COMET meetings are held annually and attended by international COS developers and other stakeholders to discuss different approaches to COS development and to learn from each other. The purpose of the workshops on including patients was to start to explore what principles, methods and strategies COS developers might want to consider when seeking patient input into the development of a COS. This article describes the discussions that took place during three interactive workshops on ways to include patients in COS development, and to make sure that COS really do reflect the perspectives of patients.

### How were the workshops organised and facilitated and who took part?

The three “Involving patients in core outcome set development: identifying the challenges and potential solutions” workshops were organised as part of the COMET Initiative meetings in Manchester (2013), Rome (2014) and Calgary (2015). The workshops were open to all COMET delegates who selected a preferred workshop from a list of available workshops. A total of 115 patients, researchers, clinicians and other stakeholders from 11 different countries registered for the “Involving Patients” workshops. We did not take a register of those who actually attended the workshops, or enquire whether attendees were patients, carers, researchers, clinicians etc., so we cannot give further details.

Before the workshops delegates received a brief description of the workshops emphasising the importance of including patients in COS development. The description also explained that workshops aimed to raise awareness of the challenges in including patients in COS development and to explore potential solutions, rather than ‘to teach’ participants ‘correct’ ways to do this. Each of the three workshops lasted 90 min and began with a 5–10 min presentation to set the scene. Delegates then organised themselves into breakout groups comprising approximately 4–6 individuals to discuss the challenges and solutions. A group of three or four patient facilitators circulated around each of the breakout groups to prompt discussion; each workshop had a different group of patient facilitators. A plenary session lasting approximately 20 min at the end of each workshop enabled the breakout groups to share ideas and experiences. The workshops were designed jointly by Heather Bagley (HB) and Bridget Young (BY), who also facilitated the workshops. The patient facilitators were recruited through various routes, including adverts on the UK National Institute for Health Research People in Research website [16] and through internet searches and personal contacts. All had experience of PPI in research and interests in COS, although very few had direct experience of COS development.

### What topics were discussed?

Before the workshops HB and BY sent briefing notes to the patient facilitators to support them in their roles. The notes listed the five main questions (see questions 1–5 below) to structure the breakout discussions, as well as follow up prompts to help facilitators to stimulate discussion. Copies of the COMET plain language summaries on COS and Delphi exercises were also made available to the patient facilitators (see http://www.comet-initiative.org/resources/PlainLanguageSummary). HB and BY met with the patient facilitators before each of the workshops to clarify the aims and discuss how the sessions would be run. The workshops evolved over time. For example, after the first workshop the briefing notes were refined to steer future discussion away from simply revisiting general guidance on patient and public involvement in research (which is increasingly available, for example, from INVOLVE [17] in the UK) and more towards the particular challenges of seeking patient input in COS development. The subsequent workshops also aimed to focus discussion on the challenges in COS studies of both: a) patient *participation* (i.e. where patients contribute data) and b) patient *involvement* (i.e. where patients take on research partner roles). At the end of each workshop we collected notes from breakout groups. We used these and some notes that a facilitator had made on each of the plenaries to prepare summaries of the discussions in each workshop. These summaries were checked by HB and BY and have informed the preparation of this article. Some of the patient facilitators also reviewed and commented on drafts of this article and we have listed their names in the acknowledgments section. In the sections that follow we take each of the five main questions in turn and outline the discussions that took place across the three workshops in response to these questions.

### Question 1: what principles and practicalities are important to consider in accessing and sampling patients?

Delegates pointed to the need to distinguish between including patients as participants in COS development and including them as research partners in the process of planning, running and disseminating a COS study. Groups discussed how COS developers may find the patient participant role best suited to individuals who can offer a personal experience of the condition at hand, whereas the patient partner role might be more suited to those who, for example, have knowledge and skills relevant to providing a patient perspective on the design and delivery of a COS study. Delegates felt that training and support should be provided for patient partners in COS development so that they are in a position to give meaningful and credible input. However, for some delegates the idea of formally training patient participants in COS development was controversial due to fears that it might influence or undermine the patient voice. It was nevertheless recognised that patient participants would need some form of support, particularly for ‘remote’ methods of COS development, such as online or postal Delphi surveys, that do not involve face-to-face or telephone contact. Potential for bias in COS development from conflicts of interest arising from sources of funding for certain organisations or activities, were discussed. Delegates felt that these could apply equally to patients, researchers, clinicians and other stakeholders, and so should be managed similarly across these groups.

There was considerable uncertainty regarding questions such as “how many patient participants are needed” in COS development or “what proportion of patients relative to other stakeholders should be included”. It is particularly important to gain a diversity of perspectives when patients and other stakeholders participate in COS development. There is currently little guidance on how many stakeholders are needed in total, or on the size and composition of each stakeholder group relative to the others, but there are indications that these factors can influence what outcomes are rated as important [18, 19]. We intend to explore these issues in future workshops. In contrast there was strong agreement on the importance of the principles of inclusivity and fairness for guiding COS development. This was reflected in extensive discussions on how to address the practicalities of making COS development activities accessible to patient participants and research partners (e.g. accommodating commitments to work, school and clinic appointments) and sensitive to the range of needs that particular groups might have arising from their illness, condition or social circumstances (e.g. need for COS development materials to be in different formats). These considerations also extended to the choice of routes for accessing patients as participants. One group suggested that community outreach and the technique of maximum variation sampling (whereby patients are sampled to include those from a wide range of backgrounds and with a wide range of relevant experience) might help in achieving diversity. Others focussed on the trade offs between accessing patient participants via health clinics versus patient organisations. In countries with publically funded healthcare systems like the UK, health clinics tend to be used by most patients and so may help in ensuring diversity. Patient organisations may in theory be open to all, yet in practice these might be accessed by a relatively narrow spectrum of patients. Social media was identified as another option for accessing patients, yet there are indications that social media samples lack diversity [20]. As COS development aims to produce generalizable knowledge, it is usually regarded as a research activity. Therefore, the need to seek ethical approval for all participants, regardless whether they are patients, clinicians or other stakeholders, was also emphasised.

### Question 2: what principles and practicalities are important in selecting a method for patient input in COS development?

It was acknowledged that different types of methods such as surveys, interviews, focus groups and consensus meetings may be needed and that COS development would usually comprise several different stages. Delegates advised that it was essential for COS developers to make sure the methods suited the needs and preferences of the particular patient groups whose input was being sought and the sensitivity of the topic. Involving patients from an early stage as partners in COS study development was regarded as crucial in making sure both the method and the mode of patient participation in COS development (e.g. whether face-to-face, online, by phone etc.) were suitable. Some groups favoured methods of participation such as qualitative interviews that allowed two-way conversations over Delphi surveys, particularly at the start and end of the process. Delegates who favoured qualitative methods thought these were good for capturing the complexity of the patient perspective, the language that patients used and understanding why some outcomes mattered more than others. Some delegates also commented that Delphi surveys were intimidating for patient participants. However, others pointed out that Delphi surveys could help to widen patient participation and promote transparency, and by offering anonymity, Delphi surveys helped to minimise the influence of power differentials between different stakeholders. Concerns were voiced about the long number of outcomes included in some Delphi surveys that patient participants would have to go through and score, and it was thought that this could be off putting for many. When working with patient groups such as children, people with learning disabilities, cognitive impairments or brain injuries, delegates advised that care was needed to avoid conflating the perspectives of patient and carer, and warned of how carers can easily ‘drown out’ the perspectives of patients.

### Question 3: what are the best ways of explaining the concept of a ‘core outcome set’ to patients and what types of questions can be used to elicit their perspectives?

The extent to which patients need a detailed explanation of COS will depend on the methods used to seek their input and whether they are acting as participants or as research partners. An advantage of qualitative interviews or focus groups for seeking the input of patient participants is that these methods allow patients to ‘set the agenda’ and talk about their experiences as they would in everyday life. This means that patient participants can take part meaningfully in COS development without needing a detailed understanding of what an outcome is, or the reasons why COS are needed [21]. Other types of input to COS development, such as participation in Delphis or consensus meetings and patient partner roles, require patients to engage more directly with concepts such as outcomes and research processes. Delegates felt strongly that patient participation within Delphis and consensus meetings needs to be meaningful and on an equal footing with other stakeholders. It follows that COS developers need to ensure that COS concepts, and methods of COS development such as Delphi studies, are explained in ways that patient participants can understand, yet without unduly influencing their responses.

Delegates advised that patient research partners had an important role in developing clear explanations of COS and associated concepts. They recognised how challenging it could be for COS developers, who were familiar with the concepts, to convey these, particularly when the concepts were far removed from the experience of most patients. In explaining COS to patients, delegates advised developers to focus on the overall goal, that is, how COS would benefit patients. Simple analogies, such as the impossibility of comparing ‘apples and pears’, were thought to be useful in explaining COS, and some delegates felt it might also be helpful for patients, particularly those in partner roles, to learn about systematic reviews and clinical trials to really understand why COS are needed. The importance of being transparent about the goal of identifying only ‘core’ outcomes was emphasised, with delegates commenting on the difficulty that patients and other participating stakeholders might otherwise have in ‘letting go’ of certain outcomes if this was not clear from the outset.

Linked to this, delegates pointed to some fundamental points of tension between the perspectives of patients and those of researchers or clinicians, and the challenges these different perspective posed for COS development. One delegate astutely observed that many patient participants and partners might understandably assume that researchers had ‘worked out what to measure years ago’ and pointed to how patients might be bewildered on first hearing that this is not the case. Similarly, when asked what is most important to them, delegates anticipated that some patient participants might respond by saying “a cure” and that nothing else really mattered to them. Delegates felt that COS developers needed to be able to respond to the other issues and queries that COS development might raise for patient participants and partners. For example, it is natural for patients to want to be cured. Yet, knowing that cure is a very distant prospect for many chronic illness, it is natural for COS developers to want to focus on more achievable outcomes. Developers need to be prepared to acknowledge the importance of cure to patients, while supporting them to consider what other outcomes matter and without inadvertently influencing them.

Methods such as online or postal Delphis were thought to require particularly careful preparation if these were to make sense to patient participants, and delegates again pointed to how patient research partners could help with developing the content and meaningfulness of items and fine-tuning the language used. As we elaborate below, delegates felt that face-to-face methods of consensus development called for first-rate facilitation skills, as well as careful preparation to ensure that patient participants were well supported. Delegates suggested a number of questions to elicit patient perspectives on what outcomes are important. These included: ‘what is the most limiting aspect of your condition’, ‘what would you hope for from a new treatment’, ‘what sort of things would you think about in making a decision about what treatment to take’ or ‘what sorts of changes in your symptoms/condition/life would tell you that a treatment was actually helping you’.

### Question 4: How can we maintain the input of patients over time?

While interviews or focus groups often require input from patient participants on only one occasion, other types of input such as patient participation in Delphi surveys and patient partner roles, require input on two or more occasions. So, maintaining the input of patients over time either as participants or partners is vital. Delegates thought that careful planning at an early stage was key here, and reiterated the importance of ensuring that the methods, design and mode of participation were suited to the patient group of interest. Managing expectations from the outset about timescales and keeping patient participants and partners informed of progress were seen as other important ways to guard against drop out or disengagement from the process. Beyond this, delegates thought that attending to common courtesies could go a long way to maintaining the input of patients as both participants and partners. These included building rapport with patients, showing appreciation for their contributions, reimbursing costs and ensuring that feedback was provided and written in plain language. Delegates also advised COS developers to consider what might incentivise patient participants to remain over the course of a project. For some participants this might entail doing the COS quickly and minimising the gap between Delphi rounds. The offer of payments or vouchers were thought to be helpful in maintaining patient input, but delegates also pointed to how building a sense of curiosity and excitement about COS development, as well as a sense of ownership of the process, might also help to maintain interest over time.

### Question 5: should patients be brought together with other stakeholders to achieve consensus? if so, how should this be done?

Delegates differed in their views about whether patient participants should be brought together in face-to-face meetings with other participating stakeholders to achieve consensus, or whether it was best for patients to meet separately. Those who favoured a separate approach were concerned about more powerful voices dominating over patients. Those who favoured bringing patient participants together to meet with other stakeholders felt it was important to avoid assuming that patients would necessarily be dominated by other groups and commented that if meetings provided an enabling environment and strong support and facilitation, patients would be capable of putting their perspectives across. Achieving an enabling environment in consensus meetings was thought to depend on good preparation, and crucially, on the skills of the facilitator. Delegates tended to favour the idea of consensus meetings being facilitated by someone who was an expert in facilitation, rather than by an expert in the particular condition or treatment. A few mentioned the possibility of having a patient partner and researcher co-facilitate meetings. Linked to this, delegates raised questions about how divergences between the perspectives of patients and other participating stakeholders would be reconciled in practice. They counselled that all parties might feel uncomfortable when their opinions were challenged. Nevertheless, they were clear that all participating stakeholders needed to be genuinely open to challenge, prepared to explain the reasons behind their opinions and to reconsidering their opinions.

## Conclusions

Across the three workshops we were struck by delegates’ enthusiasm for including patients as participants and partners in COS development and their eagerness to do so in meaningful, transparent and inclusive ways. This was sometimes accompanied by frustration that there was no straightforward set of steps to follow for seeking patients’ input into COS. Given the many different contexts in which COS developers work, there are questions about whether such a set of steps is achievable or desirable. However, it is hoped that guidance and resources will eventually be produced, which COS developers can then adapt to ensure that COS development is meaningful and suited to the patient group they are working with. Delegates felt that key to ensuring this is recognising that having patient participation in COS development alone is not enough, and that patients will also need to be involved as partners in the design and implementation of COS development studies.

Methodological work will be important to understand the COS development process from the perspective of patient participants and partners and how the process may be improved for them. Such work might usefully explore how the input of patients is currently being sought in COS studies, which ways of seeking their input work best for different patient groups or at different stages of the COS development process, and what training or support would be helpful to patients and COS developers. Work is also needed to further understand the distinctive challenges of COS development, compared to other research activities. Given that COS development can seem far removed from the concerns of patients, not least among these challenges is winning the engagement of patients as participants and partners. Developers also face distinctive challenges in ensuring COS are relevant across different countries and in bringing multiple stakeholder groups together to achieve consensus. In recognition of the vital role of patients in identifying solutions to these challenges, the COMET Initiative has established the PoPPIE (People and Public Participation, Involvement and Engagement) Working Group. PoPPIE’s international membership will be leading COMET’s public participation, involvement and engagement activities with the goal of making sure patients have a real say in COS and that the development of COS is meaningful to them.

## Abbreviations

COS, core outcome set(s); PPI, patient and public involvement

## References

[1] Williamson PR, Altman DG, Blazeby JM, Clarke M, Devane D, Gargon E, Tugwell P. Developing core outcome sets for clinical trials: issues to consider. Trials. 2012;13(1):132. 10.1186/1745-6215-13-132 PMC3472231 22867278

[2] Rimer J, Dwan K, Lawlor DA, Greig CA, McMurdo M, Morley W, Mead GE. Exercise for depression. Cochrane Database Syst Rev. 2012;7(CD004366). doi:10.1002/14651858.CD004366.pub4. 10.1002/14651858.CD004366.pub5 22786489

[3] Chalmers I, Bracken MB, Djulbegovic B, Garattini S, Grant J, Gülmezoglu AM, Howells DW, Ioannidis JP, Oliver S. How to increase value and reduce waste when research priorities are set. Lancet. 2014;383(9912):156–65. 10.1016/S0140-6736(13)62229-1 24411644

[4] Chalmers I, Glasziou P (2009). Avoidable waste in the production and reporting of research evidence. Obstet Gynecol.

[5] Gargon E, Gurung B, Medley N, Altman DG, Blazeby JM, Clarke M, Williamson PR. Choosing important health outcomes for comparative effectiveness research: a systematic review. PLoS One. 2014;9(6):e99111. 10.1371/journal.pone.0099111 PMC4059640 24932522

[6] COMET Initative-Core outcome set plain language summary—involving patients and the public in improving research [http://www.comet-initiative.org/assets/downloads/COMET%20Plain%20Language%20Summary%20v4.pdf]. Accessed 5 July 2016.

[7] Kirwan JR, Minnock P, Adebajo A, Bresnihan B, Choy E, De Wit M, Hazes M, Richards P, Saag K, Suarez-Almazor M. Patient perspective: fatigue as a recommended patient centered outcome measure in rheumatoid arthritis. J Rheumatol. 2007;34(5):1174–7. 17477482

[8] Sinha IP, Gallagher R, Williamson PR, Smyth RL (2012). Development of a core outcome set for clinical trials in childhood asthma: a survey of clinicians, parents, and young people. Trials.

[9] Gorst SL, Gargon E, Clarke M, Blazeby JM, Altman DG, Williamson PR. Choosing Important Health Outcomes for Comparative Effectiveness Research: An Updated Review and User Survey. PloS One 2016;11(1). 10.1371/journal.pone.0146444 PMC4718543 26785121

[10] Harman NL, Bruce IA, Kirkham JJ, Tierney S, Callery P, O’Brien K, Bennett AM, Chorbachi R, Hall PN, Harding-Bell A. The importance of integration of stakeholder views in core outcome set development: Otitis media with effusion in children with cleft palate. PLoS One. 2015;10(6):e0129514. 10.1371/journal.pone.0129514 PMC4483230 26115172

[11] Keeley T, Khan H, Pinfold V, Williamson P, Mathers J, Davies L, Sayers R, England E, Reilly S, Byng R. Core outcome sets for use in effectiveness trials involving people with bipolar and schizophrenia in a community-based setting (PARTNERS2): study protocol for the development of two core outcome sets. Trials. 2015;16(1):47. 10.1186/s13063-015-0553-0 PMC4334396 25887033

[12] Tong A, Manns B, Hemmelgarn B, Wheeler DC, Tugwell P, Winkelmayer WC, van Biesen W, Crowe S, Kerr PG, Polkinghorne KR. Standardised outcomes in nephrology–haemodialysis (SONG-HD): study protocol for establishing a core outcome set in haemodialysis. Trials. 2015;16(1):364. 10.1186/s13063-015-0895-7 PMC4543451 26285819

[13] Waters A, Tudur Smith C, Young B, Jones TM (2014). The CONSENSUS study: protocol for a mixed methods study to establish which outcomes should be included in a core outcome set for oropharyngeal cancer. Trials.

[14] Whitehead L, Perkins G, Haywood K (2015). ‘Getting back to normal’: using patients’ lived experience to inform a core outcome set for cardiac arrest clinical trials. Trials.

[15] Keeley T, Williamson P, Callery P, Jones L, Mathers J, Jones J, Young B, Calvert M. The use of qualitative methods to inform Delphi surveys in core outcome set development. Trials. 2016;17(1):1. 10.1186/s13063-016-1356-7 PMC4855446 27142835

[16] National Institute for Health Research-People in Research [(http://www.peopleinresearch.org/)]. Accessed 5 July 2016.

[17] INVOLVE [http://www.invo.org.uk/]. Accessed 5 July 2016.

[18] Avery K, Chalmers K, Whale K, Blencowe N, Macefield R, Brookes S, Metcalfe C, Blazeby J. The importance of stakeholder selection in core outcome set development: how surveying different health professionals may influence outcome selection. Trials. 2015;16 Suppl 2:47.

[19] Macefield R, Blencowe N, Brookes S, Jacobs M, Sprangers M, Williamson P, Blazeby J. Core outcome set development: the effect of Delphi panel composition and feedback on prioritisation of outcomes. Trials. 2013;14 Suppl 1:77.

[20] Gargon E, Altman DG, Blazeby JM, Clarke M, Williamson PR (2015). COMET IV meeting summary. Trials.

[21] Mathers J, Keeley T, Jones L, Calvert M, Williamson P, Jones J, McMullan C, Wright S, Young B. Using qualitative research to understand what outcomes matter to patients: direct and indirect approaches to outcome elicitation. Trials. 2015;16 Suppl 2:O39.

[22] Cochrane Consumer Network-What is a systematic review? [http://consumers.cochrane.org/what-systematic-review]. Accessed 5 July 2016.

[23] Peninsula Cerebra Research Unit-Research Summary [http://www.pencru.org/media/universityofexeter/medicalschool/subsites/pencru/pdfs/Peer_qualitative_study_plain_language_summary.pdf]. Accessed 5 July 2016.

[24] COMET Initative-Delphi plain language summary [http://www.comet-initiative.org/assets/downloads/Delphi%20plain%20language%20summary%20for%20COMET%20website.pdf]. Accessed 5 July 2016.

